# Botryoid Rhabdomyosarcoma of the Uterine Cervix in a Young Adult: Diagnostic Challenge and Aggressive Clinical Course

**DOI:** 10.7759/cureus.109612

**Published:** 2026-05-25

**Authors:** Kelly Haag, Bilane Daher, Nora Naqos, Othmane Zouiten, Mohamed El Fadli, Rhizlane Belbaraka

**Affiliations:** 1 Medical Oncology, Mohammed VI University Hospital, Marrakesh, MAR

**Keywords:** adult rhabdomyosarcoma, botryoid rhabdomyosarcoma, case report, rare cancer, uterine cervix

## Abstract

Rhabdomyosarcoma of the cervix is an exceptionally rare malignancy in adults, typically presenting in pediatric populations. We report the case of a 26-year-old woman initially managed for a presumed benign endocervical polyp who subsequently developed rapidly progressive botryoid rhabdomyosarcoma of the uterine cervix. Initial histological misdiagnosis delayed appropriate treatment, and despite radical surgery, the patient experienced early recurrence with peritoneal dissemination. This case emphasizes the diagnostic challenges posed by the rarity of this tumor in adults, the importance of comprehensive immunohistochemical evaluation in ambiguous cervical lesions, and the aggressive biological behavior characteristic of rhabdomyosarcoma in adults. Confirmatory molecular testing for characteristic translocations should be considered to guide prognosis and therapy selection.

## Introduction

Rhabdomyosarcoma (RMS) represents the most common soft tissue sarcoma in children but is exceedingly rare in adults, accounting for less than 1% of adult malignancies [1]. Botryoid RMS, a subtype of embryonal RMS characterized by its grape-like polypoid architecture, typically arises in hollow organs such as the vagina, bladder, and nasopharynx in children [2,3]. Cervical involvement in adults is particularly uncommon. The diagnostic challenge posed by cervical RMS in adults stems from its rarity, leading to initial misdiagnosis as benign polyps or other more common gynecological conditions [2]. Furthermore, the poor prognosis associated with adult RMS, particularly at unusual anatomical sites, necessitates prompt recognition and multimodal therapy [4]. We present this case to highlight a critical diagnostic pitfall in the evaluation of polypoid cervical lesions in young adults, where initial misdiagnosis as a benign polyp led to significant treatment delay. This report further aims to contribute to the limited literature on adult cervical botryoid RMS by documenting its aggressive biological behavior and the therapeutic challenges encountered when optimal multimodal treatment sequencing is compromised.

## Case presentation

In August 2025, a 26-year-old nulligravid woman with no significant medical, surgical, or family history presented at an outside institution with a three-week history of a painless polypoid mass protruding through the cervical os to the vulva, associated with minimal intermittent vaginal bleeding. The lesion was partially excised, and histological examination suggested a benign endocervical polyp. No further intervention was recommended at that time.

**Figure 1:**
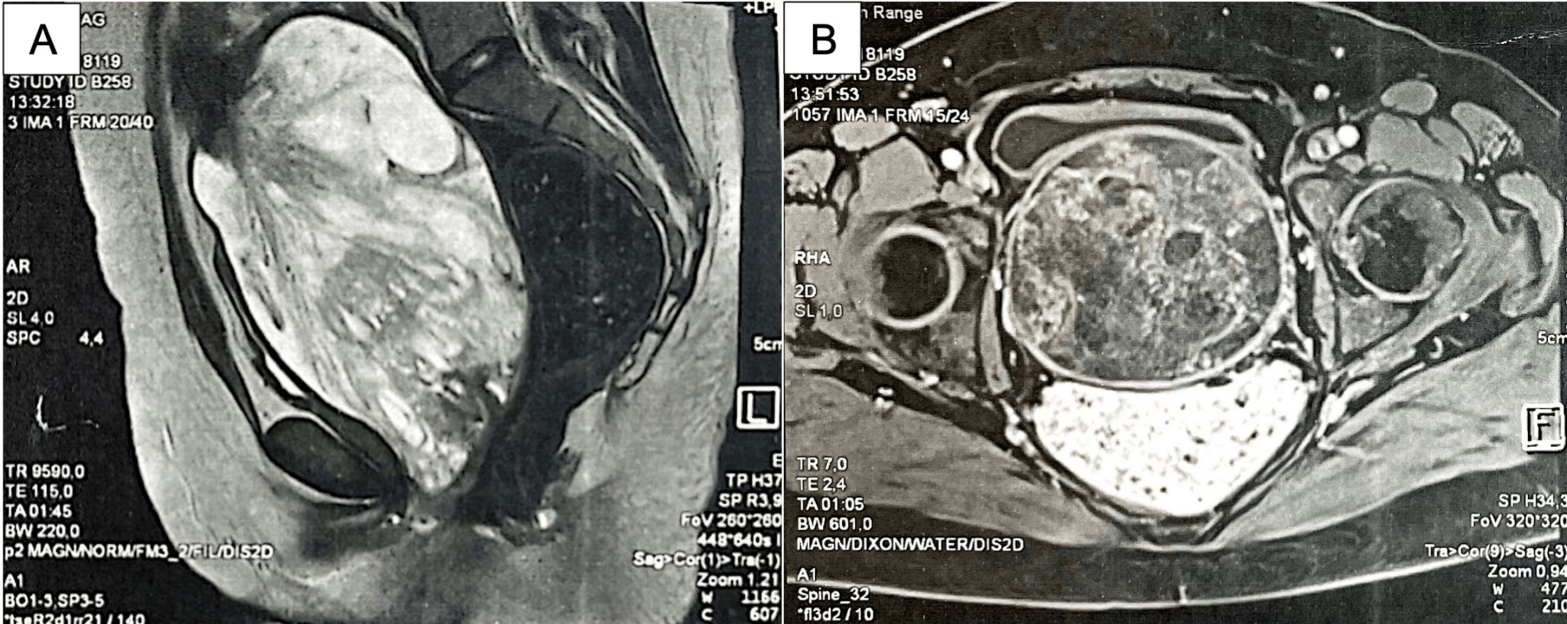
Pelvic magnetic resonance imaging demonstrating a large cervicovaginal mass. (A) T2-weighted sagittal sequence showing an 18 cm heterogeneous mass of cervical origin with mixed solid and myxoid components, extending inferiorly through the vaginal canal and exerting significant mass effect on adjacent pelvic structures. (B) Axial T1-weighted Dixon water sequence at the level of the mid-pelvis confirming the solid-cystic heterogeneous internal architecture of the tumor with well-defined encapsulation

Over the subsequent weeks, the patient developed progressive pelvic pain, leucorrhea, and vulvar protrusion of tissue masses, accompanied by constitutional symptoms including weight loss and fatigue. Twelve weeks after the initial polyp excision, a pelvic magnetic resonance imaging (MRI) revealed a large, well-circumscribed, encapsulated, ovoid abdominopelvic midline mass centered on the cervicovaginal region, measuring 180×110×100 mm (craniocaudal×anteroposterior×transverse) (Figure 1). The mass demonstrated a mixed solid-cystic architecture. The solid component was heterogeneous in signal on T1-weighted images and intermediate to hyperintense on T2-weighted sequences, with restricted diffusion and heterogeneous enhancement following gadolinium administration. The cystic component was multiloculated, containing multiple septations that appeared hypointense on T1-weighted sequences and hypointense to isointense on T2-weighted sequences, with restricted diffusion and post-contrast enhancement. There was no evidence of invasion of the bladder, rectum, or pelvic sidewalls.

Tumor markers, including beta-human chorionic gonadotropin (β-hCG), cancer antigen 125 (CA125), and alpha-fetoprotein (AFP), were within normal limits (Table 1).

**Table 1:** Blood tumor marker value β-hCG: beta-human chorionic gonadotropin; CA125: cancer antigen 125; AFP: alpha-fetoprotein

Tumor markers	Value	Normal range
CA125	16.37 UI/mL	0-35 UI/mL
AFP	2.73 ng/mL	0-9 ng/mL
β-HCG	0.36 mUI/mL	0-5 mUI/mL

**Figure 2:**
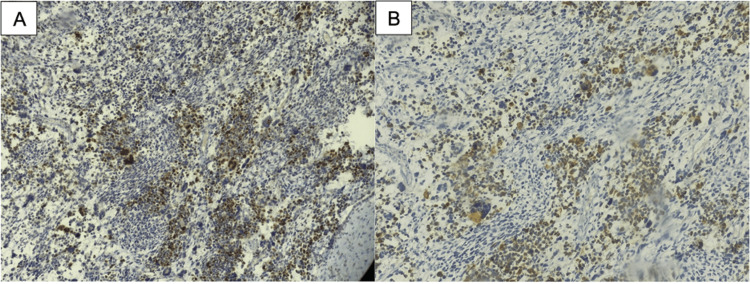
(A) Myogenin- and (B) desmin-positive staining on immunohistochemistry

Biopsy of the mass revealed undifferentiated malignant proliferation, and immunohistochemical analysis demonstrated diffuse expression of myogenin (Figure 2A) and desmin (Figure 2B), with a Ki-67 proliferation index of 80%. Staining for CK7, smooth muscle actin, and estrogen receptor was negative. These findings were consistent with alveolar RMS.

**Figure 3:**
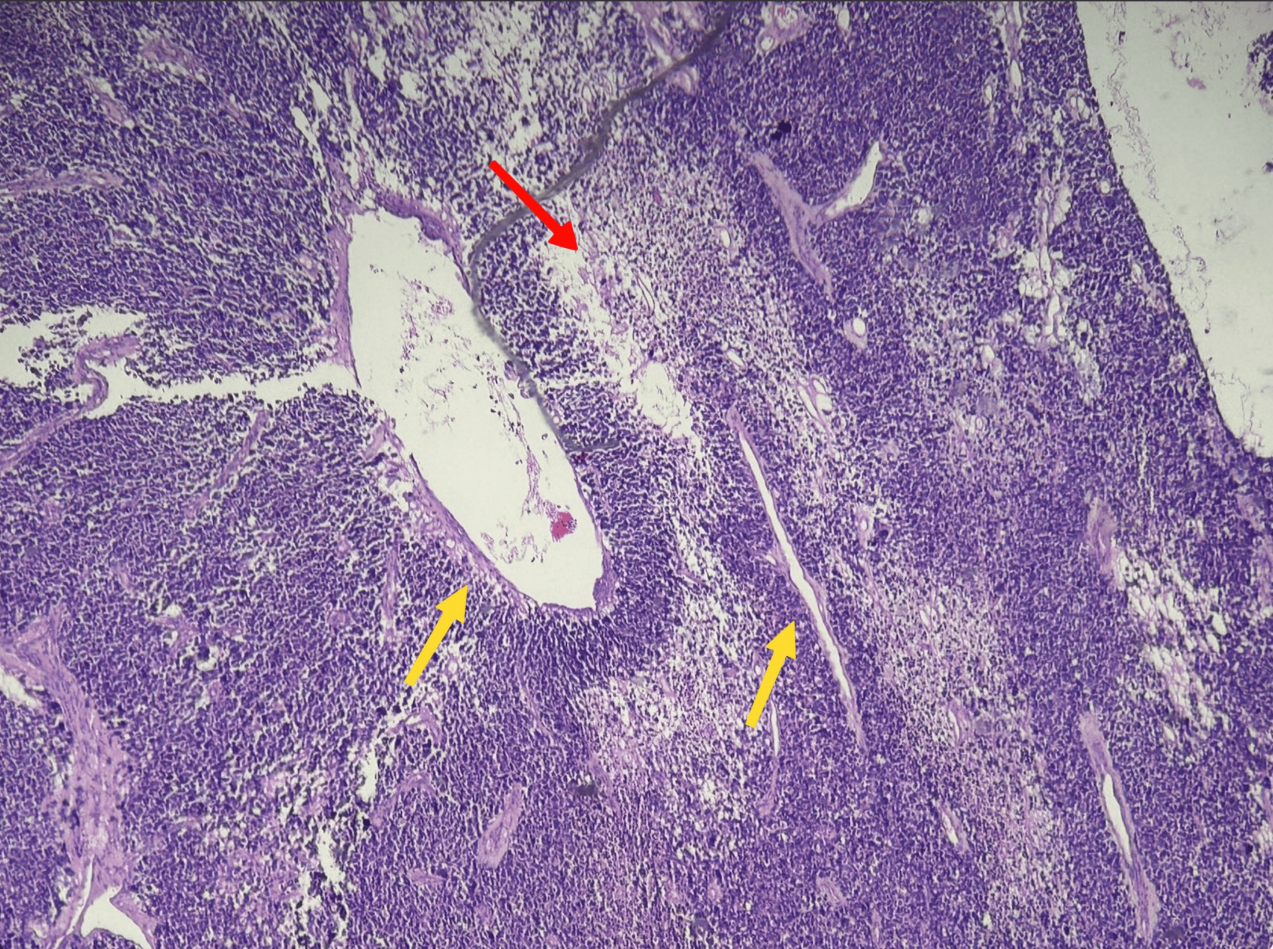
Hematoxylin and eosin staining showing primitive mesenchymal cells, polypoid architecture (surface epithelium overlying the stromal proliferation), cambium layer (dense hypercellular zone of primitive cells immediately beneath the surface epithelium, yellow arrows), alternating hypercellular zones (dark purple areas) containing primitive small round cells, and hypocellular zones with myxoid/edematous stroma (pale pink/white areas, red arrow)

The patient was referred to our institution in December 2025. Upon clinical examination, she had an Eastern Cooperative Oncology Group (ECOG) performance status of 2. Gynecological examination revealed a large, friable, grape-like polypoid mass, prolapsing through the vaginal introitus with active contact bleeding. Repeat MRI showed the large solid-cystic encapsulated abdominopelvic mass centered on the cervicovaginal region with the same characteristics as previously described. There was no evidence of distant metastases on the computed tomography of the thorax and abdomen. She was urgently managed by the surgical team due to recurrent hemorrhagic episodes, complicated by anemia. Initial resection of the extruded mass was performed, followed by total hysterectomy with the preservation of the adnexae and extended tumor resection. Final histopathological examination confirmed botryoid RMS of the cervix, measuring 16 cm. The tumor exhibited characteristic polypoid grape-like architecture with histological features including a mesenchymal proliferation showing alternating hypocellular zones with myxoid stroma and dense compact areas, a hypercellular subepithelial cambium layer, and focal cartilaginous metaplasia (Figure 3). The tumor infiltrated the cervix with extension into the uterine corpus, but surgical margins were clear.

**Figure 4:**
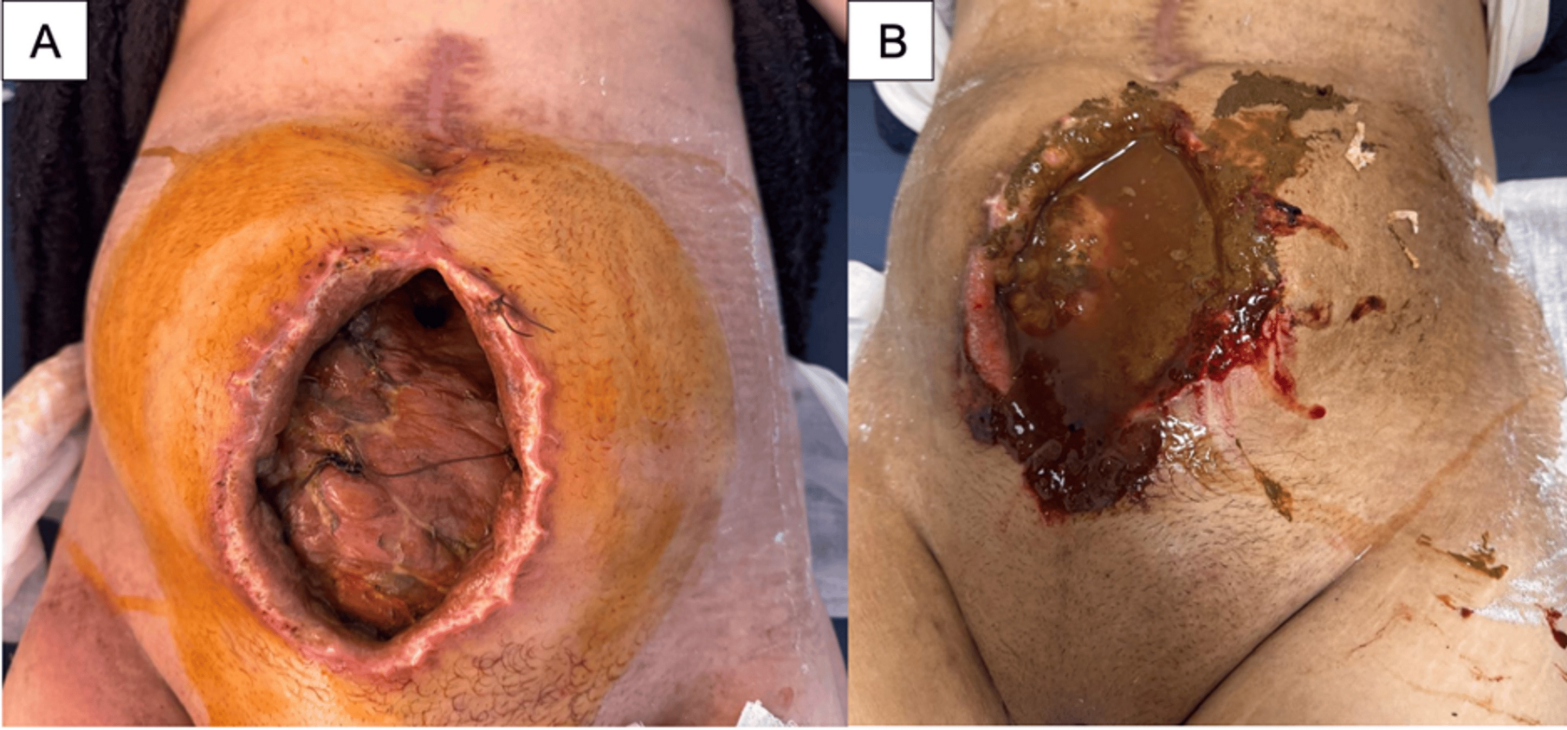
(A) Surgical wound dehiscence, exposing extensive locoregional tumor progression. (B) Fecal discharge through the surgical wound, confirming the presence of an entero-cutaneous fistula secondary to tumor enteral/colic invasion

The immediate postoperative course was complicated by local infectious complications with pelvic collections requiring surgical drainage and antibiotic therapy. Three weeks after surgery, repeat MRI revealed early tumor recurrence as an 11×9×12 cm central pelvic mass with associated peritoneal nodules. Given this rapid progression, chemotherapy was initiated with the VAC regimen: vincristine 1.5 mg/m² (days 1, 8, and 15; max single dose 2 mg), dactinomycin 45 mcg/kg (day 1; max single dose 2.5 mg), and cyclophosphamide 1,200 mg/m² (day 1), 21-day cycle with granulocyte-colony stimulating growth factor. One week after the first dose, the clinical course deteriorated with persistent suppuration, surgical wound dehiscence (Figure 4A), and the development of an enteric fistula secondary to extensive locoregional tumor progression (Figure 4B). These complications were attributed to disease progression rather than chemotherapy toxicity, though the immunosuppressive effect of chemotherapy may have impaired wound healing. Creation of a diverting stoma was deemed unfeasible due to extensive peritoneal and bowel involvement. Palliative chemotherapy was continued on the same VAC protocol. The patient is currently completing four cycles, with satisfactory overall tolerability, progressive wound healing, and a notable reduction in fistula output.

## Discussion

RMS accounts for approximately 50% of soft tissue sarcomas in children but represents only 2-5% of adult soft tissue sarcomas [4,5]. In children, the most common anatomic sites are the head and neck (approximately 40%), genitourinary tract (20-25%), and extremities (20%) [6]. In the female genital tract, the site distribution is age-dependent: vaginal RMS predominates in young children, whereas cervical involvement is more common among adolescents and premenopausal women [7].

The rarity of cervical RMS in adults contributes significantly to diagnostic delay, as clinicians may not consider malignancy when evaluating polypoid cervical lesions in young women [5,8]. Botryoid RMS characteristically presents as grape-like polypoid masses that can be mistaken for benign polyps, especially when biopsies are superficial and fail to sample deeper tissues where malignant cells concentrate [2,9].

The discordance between the initial biopsy diagnosis suggesting alveolar RMS based on immunohistochemistry and the final surgical specimen confirming botryoid RMS warrants discussion. The diagnostic confusion likely arose from the interpretation of immunohistochemical patterns on limited biopsy material. Both subtypes express myogenin and desmin, but alveolar RMS typically shows more diffuse nuclear myogenin expression [10]. The definitive diagnosis rests on the histological architecture observed in the resection specimen, which in this case demonstrated the characteristic polypoid grape-like configuration, cambium layer, and myxoid stroma of botryoid RMS. The high Ki-67 proliferation index (80% in this case) reflects the aggressive proliferative activity characteristic of high-grade RMS.

Alveolar RMS is characterized by the pathognomonic PAX3-FOXO1 or PAX7-FOXO1 fusion genes resulting from t(2;13) or t(1;13) translocations, which carry a significantly worse prognosis [10]. In contrast, botryoid RMS, a variant of embryonal RMS, generally lacks specific fusion genes and is instead strongly associated with DICER1 mutations (≥95% of uterine embryonal RMS). The presence of cartilaginous differentiation, as in this case, is particularly characteristic of DICER1-mutated botryoid RMS [2,9,11].

Adults with RMS have a significantly poorer prognosis than children, with a five-year survival rate of 26-40% compared to 60-70% in pediatric patients [5]. In this case, multiple poor prognostic indicators were present: adult age, large tumor size (16 cm), and cervicovaginal location. Early postoperative recurrence, despite negative surgical margins, further reflects the aggressive tumor biology of this tumor.

Contemporary treatment of RMS follows multimodal protocols combining surgery, chemotherapy, and radiotherapy. Current guidelines recommend initiating chemotherapy within six weeks of diagnosis, often before definitive surgery in adults with high-risk features and for tumors that are not initially resectable without significant morbidities [4,9].

However, this case deviated from optimal treatment paradigms due to initial diagnostic delay. Primary hysterectomy, although justified by the hemorrhagic emergency, deprived the patient of the potential benefit of tumor reduction through chemotherapy, which might have allowed for less extensive surgery/better local control or a better assessment of chemosensitivity.

The development of peritoneal dissemination, enteric fistula, and inability to perform palliative surgical intervention reflects advanced locoregional progression. At this stage, treatment options are limited to palliative chemotherapy.

## Conclusions

This case highlights the importance of maintaining clinical suspicion for rare malignancies when assessing atypical cervical lesions in young adults. Although uncommon in adults, botryoid RMS must be considered in the differential diagnosis of polypoid cervicovaginal masses. Accurate diagnosis and prognostic stratification rely on adequate tissue sampling, comprehensive immunohistochemical analysis, and molecular testing for characteristic translocations. Early recognition and prompt initiation of multimodal therapy guided by current pediatric protocols are essential to optimize outcomes in this challenging disease.
